# Hands-on brain slicing activity based on Gagné’s instructional model: A novel pedagogy for mastering neuroanatomical cross sections

**DOI:** 10.30476/JAMP.2020.86497.1242

**Published:** 2022-01

**Authors:** RAJASEKHAR S.S.S.N, DINESH KUMAR. V

**Affiliations:** 1 Department of Anatomy, Jawaharlal Institute of Postgraduate Medical Education and research, Puducherry – 605006, India

## Dear Editor

The key paradigm of neuroanatomy education is enabling students to unstack the complex three-dimensional anatomy of a structure into multiple two-dimensional images. Organ slicing is one such pedagogy whereby students are actively involved in slicing the organ e.g.: brain in a way resembling CT / MRI sections. This method would therefore strengthen the visuo-spatial abilities of the students while interpreting neuro-radiological images in the future. We, at our institute, have designed a hands-on brain slicing activity and we wish to document it along with the associated cognitive processing principles involved in it. 

We allotted 10 formalin fixed brain specimens to 15 students for slicing. Among them, certain students had a chance to section the brain, while others assisted and observed the sectioning. In order to provide a uniform and engaging, immersive experience to all students, we incorporated the sequential steps of Gagné’s instructional model in brain slicing activity ( 1 ). The activity was aligned with learners’ cognitive processing in order to garner optimal learning outcomes, promote retention and transfer of learning. Initially, the students were explained about their roles and the objectives of the session [Events 1 & 2 of Gagné’s model: gaining attention and informing about objectives]. Next, during the hands-on slicing, the students were asked to note the angle of knife with reference to the brain surface, and upon slicing, the photographs of sliced side and its opposite side were taken. At the same time, students were asked questions about the structures in order to stimulate recall of prior learning [Events 3 & 4 of Gagné’s model: reactivation of prior learning and presenting the content]. Then, the students were asked to make a Microsoft PowerPoint^®^ presentation individually with the pictures taken during slicing. They were instructed to stack the images according to the sequence of slicing (coronal, transverse and sagittal) and label the structures in it. This component of the module, on the one hand, would enable the students to activate the knowledge for labelling the structures and on other hands would make them identify the level at which the particular slice was taken [Events 5 & 6 of Gagné’s model: providing learning guidance and eliciting performance]. The individual presentation made by the students were assessed and then graded according to the number of labelled structures and accuracy of labelling. Based on this, feedback was given to individual students and rectifications were mentioned [Events 7 & 8 of Gagné’s model: assessing performance and providing feedback]. Once all the students had completed the task, an integrated radiological anatomy session was held where students were asked to identify the annotated structures in flashcards containing brain sections and MRI sections of the brain. This is to enhance the retrieval of visual imagery and enable the transfer of knowledge gained via the pedagogical activity [Event 9 of Gagné’s model]. 

*Javaid et al*. ( 2 ) proposed the contributing factors which potentially impair the understanding of neuroanatomy. They postulated that, when compared to extrinsic factors (such as truncated time allotted in the curriculum, and books that are written in difficult language), intrinsic factors (such as understanding the orientation of structures, visualization of structures in prosected specimens, and converting 2D visualization into 3D format) often turn out to be the likely barriers for effective neuroanatomy learning. We should also consider the fact that not all students would have satisfactory visuo-spatial abilities, and this might indirectly make them unengaged during slicing classes ( 3 ). Thus, an effective pedagogical strategy for cross-sectional neuroanatomy teaching should be centred on both psychomotor and cognitive domains of learning ( 4 ). Especially when our objective is to enable knowledge retention and transfer it to other scenarios, we need to adopt a combination of learning activities and assess the learning gain on an individual basis. 

Owing to the paucity of brain specimens in our gross anatomy laboratory, we were able to allot ten brain specimens to sectioning. This shortcoming might lead to the difference in the learning experience among the same cohort of students who participated in the slicing activity. This is because the students who were able to cut the brain specimens, i.e. involved in ‘active dissection’ may appreciate the immersive experience that slicing has to offer compared to the ‘passive observing’ counterparts, who were observing it ( 5 ). Indeed, an ideal slicing activity should engage both cognitive and psychomotor domains of learning. And unless multisensory stimulation is provided, learning will not be optimal ( 6 ). Similar to the present study, we asked to photograph the sliced part. Nevertheless, this also engaged two or three more students leaving the rest unengaged. 

To conclude, we feel that hands-on organ slicing is a valuable pedagogy for making students understand the cross-sectional neuroanatomy. However, this would not actively engage a large group of students, especially in settings where the number of the specimen is fewer than that of the students. In such situations, it is imperative to modify the activity in order to enhance the learning outcomes, and one such example is ours where we blended Gagné’s instructional model. As this model reinforces the active learning along with the internal cognitive reflection processes of students, the ultimate objective can be easily achieved.

## Conflict of Interest:

None Declared.
